# Supplementary material for: Monitoring Patients with Metastatic Hormone-Sensitive and Metastatic Castration-Resistant Prostate Cancer: A Multidisciplinary Consensus Document
Source: Cancers (Basel). 2019 Dec 1;11(12):1908. doi: 10.3390/cancers11121908 (PMC6966424; doi:10.3390/cancers11121908)
Supplement: Supplementary file 1 [file cancers-11-01908-s001.pdf]

Table S1. Consensus conference participants.

NAME		SPECIALTY	CITY
ALONGI	FILIPPO	Radiation Oncology	Verona
ALTIERI	VINCENZO	Urology	Salerno
AVUZZI	BARBARA	Radiation Oncology	Milan
BALDAZZI	VALENTINA	Medical Oncology	Empoli
BARNI	SANDRO	Medical Oncology	Treviglio
BERTONI	FILIPPO	Radiation Oncology	Brescia
BONCIARELLI	GIORGIO	Medical Oncology	Este
BORDONARO	ROBERTO	Medical Oncology	Catania
BORSELLINO	NICOLO'	Medical Oncology	Palermo
BORTOLUS	ROBERTO	Radiation Oncology	Aviano
CANTE	DOMENICO	Radiation Oncology	Ivrea
CARLINI	PAOLO	Medical Oncology	Rome
CARONE	ROBERTO	Urology	Siena
CARRINO	MAURIZIO	Urology	Naples
CARROZZA	FRANCESCO	Medical Oncology	Ravenna
CARTENI'	GIACOMO	Medical Oncology	Naples
DE LUCA	STEFANO	Urology	Torino
DE TURSI	MICHELE	Medical Oncology	Chieti
DELL'ATTI	LUCIO	Urology	Ancona
DI LORENZO	GIUSEPPE	Medical Oncology	Naples
DI MUZIO	NADIA	Radiation Oncology	Milan
DONATO	VITTORIO	Radiation Oncology	Rome
FANDELLA	ANDREA	Urology	San Donà di Piave
FANTI	STEFANO	Nuclear medicine	Bologna
FERRIERO	MARIA	Urology	Lecce
FERSINO	SERGIO	Radiation Oncology	Verona
FORNARINI	GIUSEPPE	Medical Oncology	Genova
FRATINO	LUCIA	Medical Oncology	Aviano
FREZZA	GIOVANNI	Radiation Oncology	Bologna
FUSCO	VINCENZO	Radiation Oncology	Rionero in Vulture
GASPARRO	DONATELLO	Medical Oncology	Parma
GERNONE	ANGELA	Medical Oncology	Bari
IACULLI	ALESSANDRO	Medical Oncology	Bergamo
INGROSSO	GIANLUCA	Radiation Oncology	Rome
LUCARINI	SILVIA	Radiology	Firenze
MAGLI	ALESSANDRO	Radiation Oncology	Udine
MENCOBONI	MANILO	Medical Oncology	Genova
MORELLI	FRANCO	Medical Oncology	San Giovanni Rotondo
MORLACCO	ALESSANDRO	Urology	Padua
MORTELLARO	GIANLUCA	Radiation Oncology	Palermo
MOSCA	ALESSANDRA	Medical Oncology	Novara
MUCCIARINI	CLAUDIA	Medical Oncology	Carpi
MUNOZ	FERNANDO	Radiation Oncology	Aosta
MUSIO	DANIELA	Radiation Oncology	Rome
NAGLIERI	EMANUELE	Medical Oncology	Bari
OLIANI	CRISTINA	Medical Oncology	Montecchio Maggiore
ORTEGA	CINZIA	Medical Oncology	Alba
PANARELLO	DANIELE	Urology	Genova
PASSALACQUA	RODOLFO	Medical Oncology	Cremona
PERACHINO	TOMMASO	Urology	Alessandria
PRAYER GALETTI	TOMMASO	Urology	Padova
RICOTTA	RICCARDO	Medical Oncology	Milan
ROSCIGNO	MARCO	Urology	Bergamo

SABBATINI	ROBERTO	Medical Oncology	Modena
SALVIONI	ROBERTO	Urology	Milan
SANGUINETI	GIUSEPPE	Radiation Oncology	Rome
SANSEVERINO	ROBERTO	Urology	Nocera Inferiore
SANTONI	MATTEO	Medical Oncology	Macerata
SERNI	SERGIO	Urology	Florence
SPAGNI	MATTEO	Urology	Reggio Emilia
TAVONI	FRANCESCO	Urology	Modena
TIMON	GIORGIA	Radiation Oncology	Genova
TRALONGO	PAOLO	Medical Oncology	Siracusa
TRIGGIANI	LUCA	Radiation Oncology	Brescia
TUCCI	MARCELLO	Medical Oncology	Orbassano
VESPASIANI	GIUSEPPE	Urology	Rome
ZAFFARONI	NADIA	Biology	Milan
ZATTONI	FILIBERTO	Urology	Padua
ZERINI	DARIO	Radiation Oncology	Milan
